# Semi-automatic measurements and description of the geometry of vascular tree based on Bézier spline curves: application to cerebral arteries

**DOI:** 10.1186/s12938-018-0547-8

**Published:** 2018-08-29

**Authors:** Jarosław Żyłkowski, Grzegorz Rosiak, Dominik Spinczyk

**Affiliations:** 1 Second Department of Clinical Radiology, Medical University of Warsaw, 1a Banacha, 02-097 Warsaw, Poland; 2 Faculty of Biomedical Engineering, Silesian University of Technology, Roosevelta 40, Zabrze, Poland

**Keywords:** Geometry of cerebral vessels, Computer-aided assessment of geometry of cerebral vessels

## Abstract

**Background:**

The geometry of the vessels is easy to assess in novel 3D studies. It has significant influence on flow patterns and this way the evolution of vascular pathologies such as aneurysms and atherosclerosis. It is essential to develop robust system for vascular anatomy measurement and digital description allowing for assessment of big numbers of vessels.

**Methods:**

A semiautomatic, robust, integrated method for vascular anatomy measurements and mathematical description are presented. Bezier splines of 6th degree and continuity of C3 was proposed and distribution of control points was dependent on local radius. Due to main interest of our institution, the system was primarily used for the assessment of the geometry of the intracranial arteries, especially the first Medial Cerebral Artery division.

**Results:**

1359 synthetic figures were generated: 381 torus and 978 spirals. Experimental verification of the proposed methodology was conducted on 400 Middle Cerebral Artery divisions.

**Conclusions:**

In difference to other described solution all proposed methodology steps were integrated allows analysis of variability of geometrical parameters among big number of Medial Cerebral Artery bifurcations using single application. This allows for determination of significant trends in the parameters variability with age and in contrary almost no differences between men and women.

## Background

The vessel geometry is a main feature assessed by noninvasive, three-dimensional angiographic studies such as computed tomography angiography (CTA) and MRA (magnetic resonance angiography). Its influence on the development of vascular diseases such as aneurysms and atherosclerosis has been widely studied through last decades [1–7]. New possibilities for studying vascular anatomy arise due to rising number of performed angiographies. They are performed in individuals of all ages, with normal or altered anatomy or with vascular diseases such as aneurysms and atherosclerosis [8–10]. Large numbers of cases allow analyses of geometrical differences between age and sex groups and between patients with and without given diseases. Assuming that differences in average values of various groups (sex and age dependent) in the whole population represent general trends in geometry changes in individuals we could better understand this processes and their influence on the vascular pathologies evolution. This kind of strategy is being used in the cosmology for studying galaxy evolution where it is not possible to analyze evolution of particular galaxy. In medicine we still do not have CTA or MRA series covering whole lifetime of any individual. The main requirement for this type of studies if fast and robust system for vascular anatomy measurement and digital description allowing for assessment of great numbers of vessels.

Computer aided, three-dimensional studies of vascular anatomy have been done by many authors [4–6]. When analyzing their methods and results we found that all presented systems were combined with separated modules, each for individual step of preprocessing, measurement and calculations. The vessel analyses were robust but also labor-intensive. This feature makes the application of these system in the large-number studies not practical. In our system we reduced the number of modules to two: one for measurements and data storage and one for the data analysis and collating.

The system of the description of the vessel anatomy applied by our team is not new and is based on works of Italian and Irish teams [4–10]. It is based on centerlines of vessels and allows for proper and robust description of different types of the vascular structures (both straight and diverging vessels).

The aim of this study is to propose semiautomatic, robust, integrated and fast system for vascular anatomy measurements and mathematical description studies with large number of subjects. The application of a spline curve, consisting of Bezier segment for centerlines approximation is a new concept. Smoothing and determination of curvature and torsion was a basic concept of central lines (CLs) transformation into mathematical functions. This step was achieved by transformation into Bezier splines of degree 6. The continuity of C3 degree was proposed and distribution of control points was dependent on local radius. The system was primarily used for the assessment of the geometry of the intracranial arteries, especially the first MCA division being an area of interest of our institutions.

The paper is organized as follows: in “Methods” section, useful convention and information are introduced. Then, the proposed methodology steps are presented in details (“Preprocessing the contrast enhanced CT examination”, “Analysis of the vessel cross section”, “Determination of the centerlines”, “Description of the centerlines”, “Calculation of the division zones of the vessel”, “Mathematical analysis of the centerlines”). “Materials and validation method” section describes the data set that was used and validation approach. “Results” section shows the outcomes in a different manner for both synthetic and clinical data. The obtained results are analyzed in reference to other works addressing the subject of vessel geometry in “Discussion” section. The last chapter is “Conclusion” section which summarizes results of the study.

## Methods

**Fig. 1:**
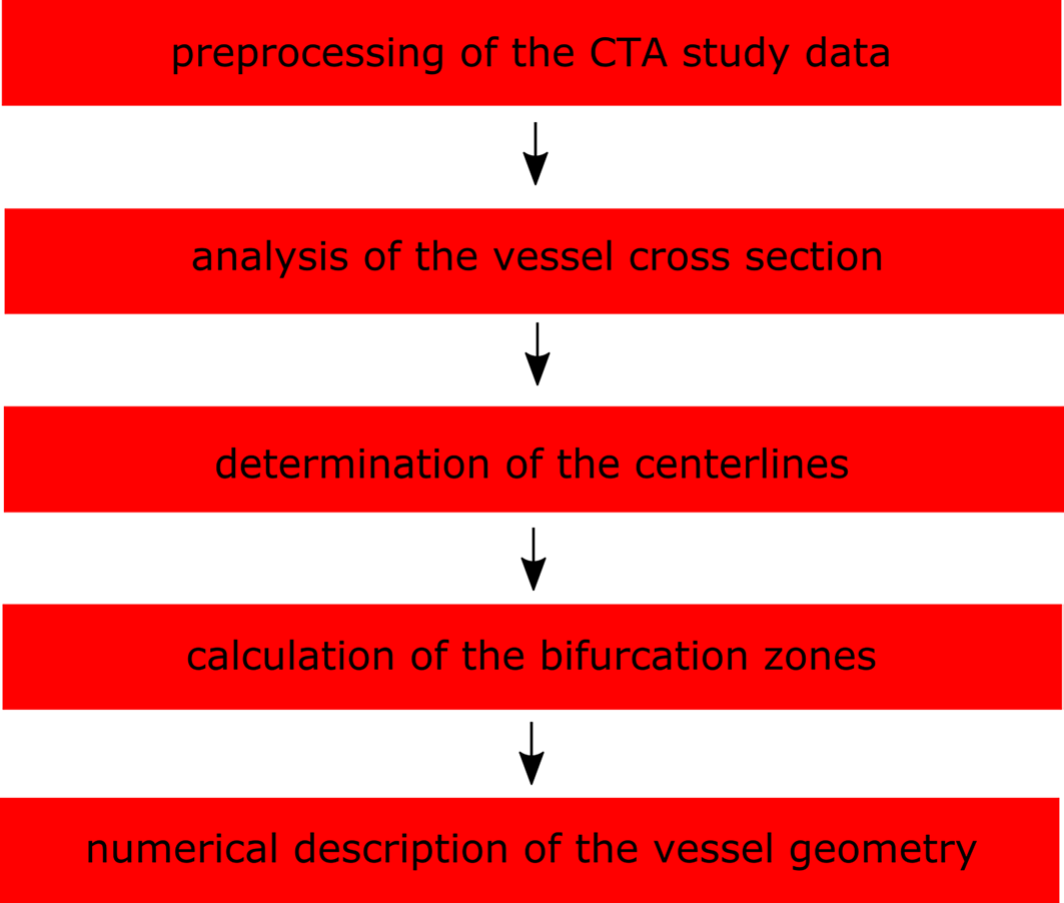
The proposed methodology steps

The methodology steps are presented in the flow chart (Fig. 1) and are described in the next paragraphs of this section: preprocessing of the CTA study data, analysis of the vessel cross section, determination of the centerlines, calculation of the division zones of the vessel and finally numerical description of the vessel geometry.

### Vessels topology

**Fig. 2:**
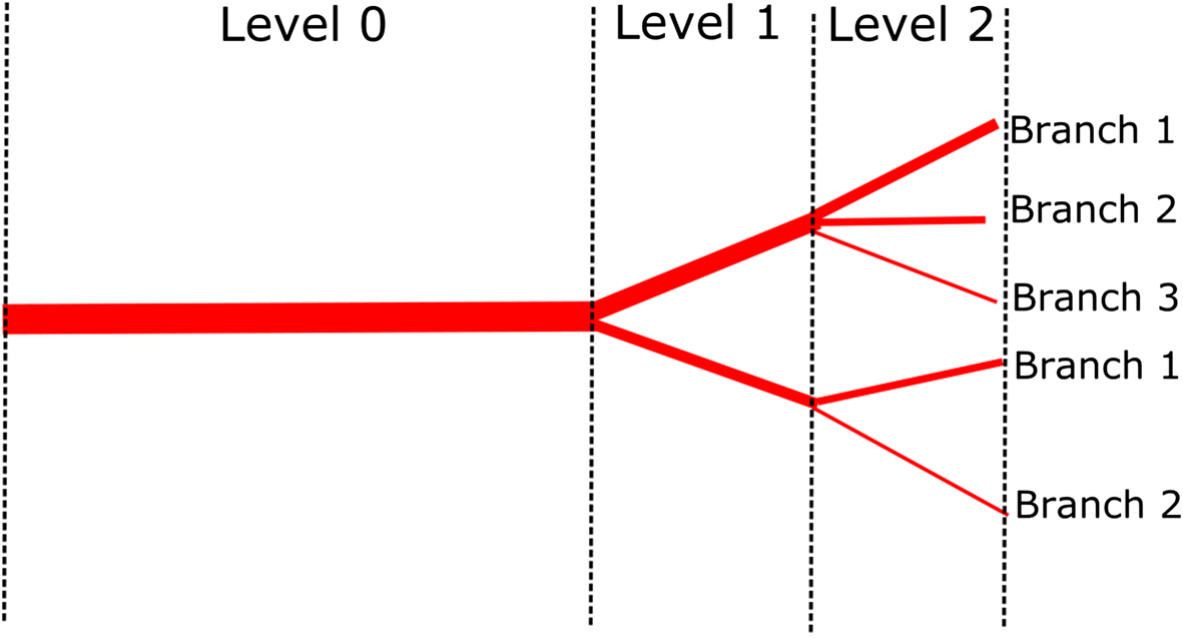
Schema of topology system defining levels and branches

System of vessels topology is shown in Fig. 2. The location in the vascular tree was described using two integers. The first one was Level zero-based index of vessel, rising distally. The vessel designation on particular Level was started from number 1. The following branches were sequentially numbered according to their declining cross section area.

### Angles and coplanarity index calculation

**Fig. 3:**
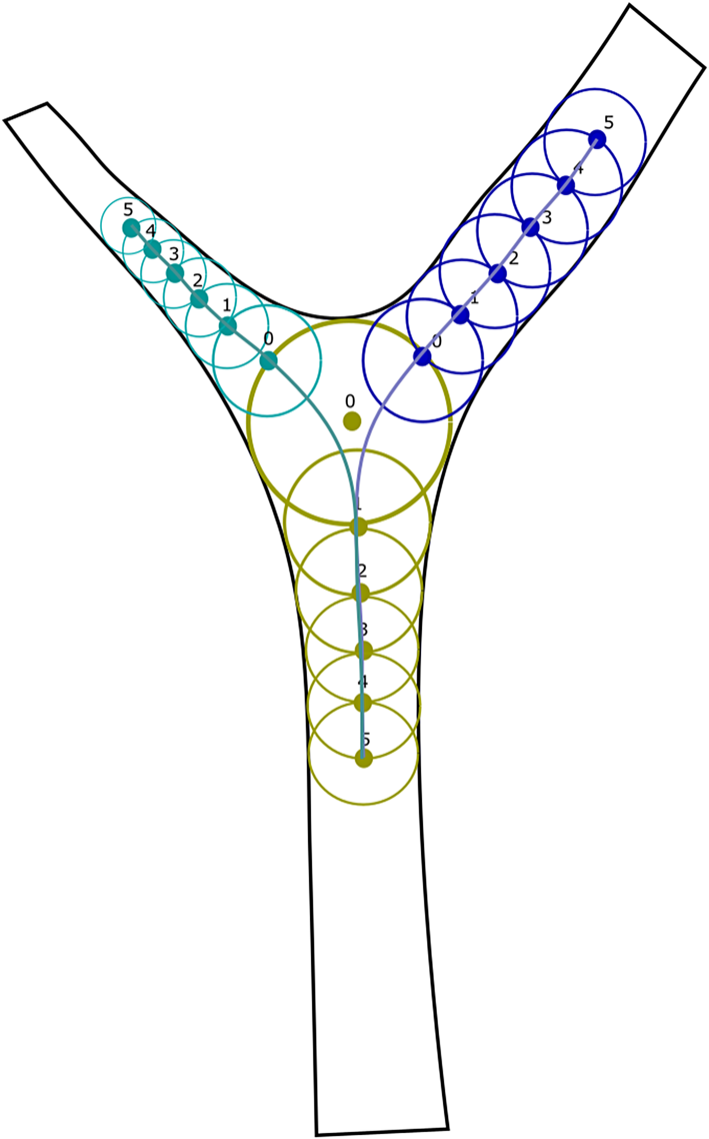
Diagram of the division zone of the vessel: $T_r$ points—green, $B_r$—blue

Numerical description of the vessels division was based on concept of the bifurcation zone. Accordingly to recent works [11–17] it was defined as the set of points on the course of the centerlines of vessels forming the division. It includes trunk points (T_r_) and branch points ($B_r$). The $T_r$ and $B_r$ points of the stem and branches are numbered from 0 to n (n $\in$ N and n > 0). The numbering of the $B_r$ correspond to the direction of the blood flow, while $T_r$ points is in the opposite direction (Fig. 3).

**Fig. 4:**
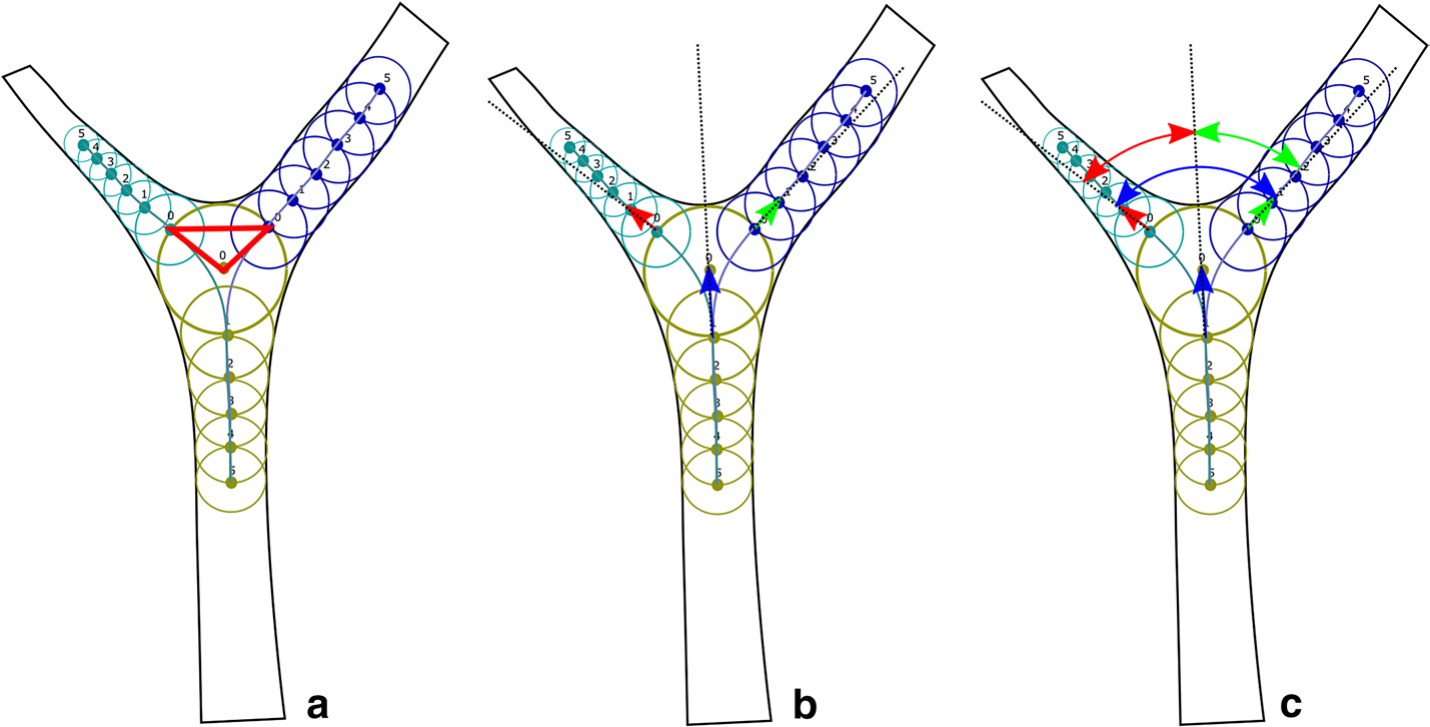
Planes, vectors and geometrical parameters of bifurcation zone. **a** Bifurcation plane (red), **b** directional vectors: trunk (blue) and vessels (red and green), **c** angles: (blue arrow), vessels angles (red and green arrows)

Determined points were used to construct planes and vectors (Fig. 4):

- Division plane (DP)—containing points 0 of each vessel engaged in the division,
- Vessel directional vectors (VDV)—vector $B_r0{-}B_r1$ and $T_r1{-}T_r0$.

Defined plane and vectors allowed for calculation of:

- Division plane normal (DPN)—vector perpendicular to DP,
- Branching angle (BA)—between VDV of both branches,
- Vessel angle (VA)—between VDV of the trunk and particular branch,
- Coplanarity index (CoI) calculated as presented in Eq. (1)
  1 $$CoI_{N} = 1 - \left| {\frac{{\angle (VDV_{N} ,DPN)}}{{\frac{\pi }{2}}}} \right|$$

### Curvature and torsion of the curve

**Fig. 5:**
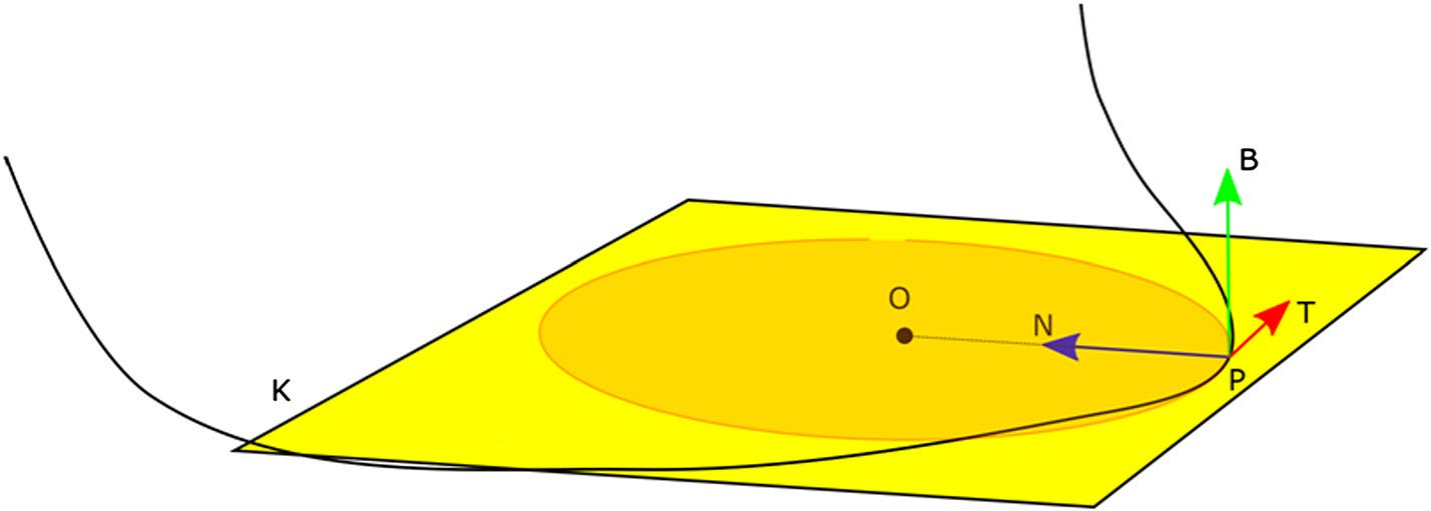
Frenet–Serret frame; where: K—spatial curve; P point on the curve; A—plane in which the curve K moves at point P; T—tangent of curve K at point T; N—normal curve K at point P; B—binormal curve K at P

The course of the curve in space is related to the concept of Frénet–Serret frame. Figure 5 shows the course of the spatial curve K. At point P, it passes in plane A after a momentary arc with center at point O. The torsion of the curve ($\tau$) at point P is defined as:

2 $$\begin{aligned} \tau _P= & {} \lim _{P'\rightarrow P}\frac{\alpha }{|P'P|}. \end{aligned}$$

where $\alpha$ is the angle between the binomial at points P′ and P. The analysis of the curvature and the approximation of the centered curve approximation in the division zone was made within the range of the arc involving the division of vessels (Arc P).

In order to determine the position on the central curve, the distance in mm was used. The calculation of the distance of the point and ($l_i$) from the beginning of the curve in mm was made discretely on the basis of the sum of the lengths of all the sections connecting the following points defining the curve and located before the point for which the position was calculated:

3 $${\L}_{i} = \sum\limits_{{n = 1}}^{i} {\left| {p_{{n - 1}} p_{n} } \right|}$$

### Preprocessing the contrast enhanced CT examination

The user sets volume of interests (VOI) in which the remaining measurements were performed. Within the VOI the resolution was raised to 0.2 mm ($\sim$ threefold typical resolution of CTA study) in each dimension and this step utilizes tricubic value approximation method. The vessels was segmented utilizing thresholding with two low levels of 100 Hounsfield Units (HU) up to 150 HU. Values of both levels were established during initial phase of the project. The lower values were chosen for strictly arterial phase studies, which was defined as enhancement of no more than 50 HU of deep Sylvian veins occupying space around MCA trunk [18, 19].

After segmentation step, calculation of maximum value of minimal distance from model edge (MOBBM) was performed in the VOI. The results of this step values were used by centerline detection algorithms. In the last step of this part of the analysis the user sets one start point on vessel level-0 and one endpoint on each vessel level-1.

### Analysis of the vessel cross section

The purpose of this stage is to automatically orient the measuring plane perpendicular to the long axis of the vessel and measure diameter (minimal, maximal and average) and vessel section area.

**Fig. 6:**
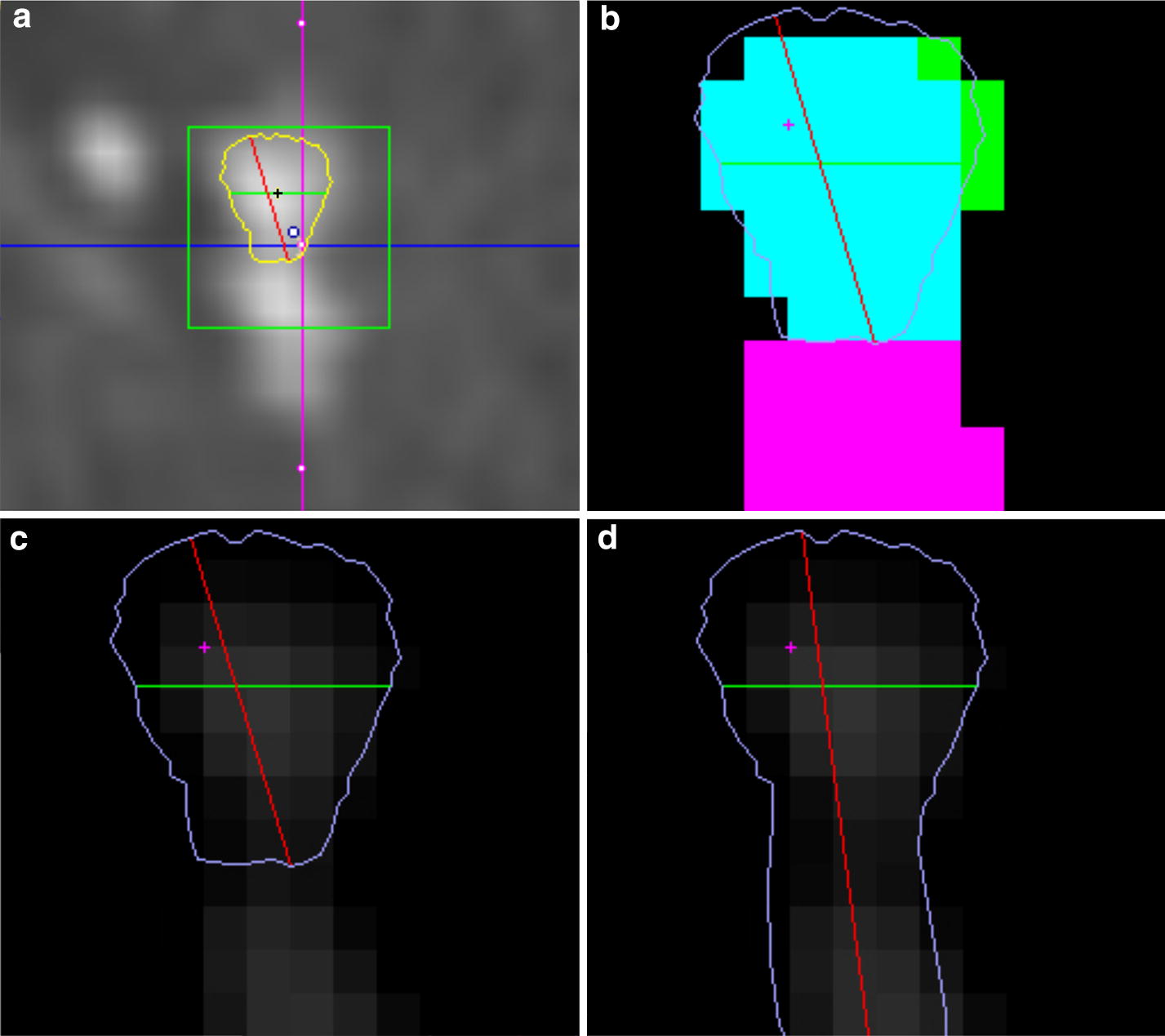
Vessel cross-section analysis: a method of tracing the vessel (**a**), the effects of subsequent algorithm steps, dividing the section into groups of points and finding the border (**b**). Effect of switching on (**c**) and off (**d**) function preserving crossing close related vessels borders

The input parameters for the method, expressed in HU, are: the range considered as the core of the vessel [$C_{min}$;$C_{max}$], lower threshold value of border area of adjacent vessels—$M_{min}$, lower threshold value of the end of the region growing process—$O_{dv}$. To accomplish this goal the following specific steps are required:

- Initialization—consists of manually entering the vessel cross-section into a rectangular area.
- Finding the geometric center of the vessel $P_{sc}$.
- Finding the border of a vessel—the algorithm uses a specific way of distributing values across the vessel. The central part of the vessel exhibits clearly higher values, which, when away from the center, initially drop slightly, and then, closer to the edge, go down rapidly. In case of neighboring vessels when moving between them, a rise of values is observed after an initial drop subsequent to crossing of the vessels edge. Images in Fig. 6c, d show differences in the result of the algorithm when detection of neighboring vessels was set to on and off respectively.
- Smoothing of the border of the vessel $P_0$—uses the local average distance from the geometric center of the vessel.

Based on the value of the input parameters, the threshold and labeling of the core region of the vessel and the center of gravity are determined. After transformation of the $P_{SC}$ to the global coordinate system, the algorithm of finding the edge of the vessel in n directions with step $2\pi /n$ is n-folded. The result of this step is obtaining a set of n 3D points describing the shape of the border. The last step was to find the extremes and the average diameter and cross-sectional area. Extremities were found by analyzing the length of the sections between the border points with indices i and n + 2, where n is the number of directions in which edge points were searched, $i\in [0,n/2)$. The cross-sectional area (PPP) was calculated as the sum of the surface area of the triangles: ($P_{sc}$, $P_{0i}$, $P_{0i + 1}$) where $i \in [0, n-1]$ and ($P_{SC}$, $P_{0n-1}$,$P_{00}$). The average diameter $\Delta$d was calculated from PPP by the formula:

4 $$\begin{aligned} \Delta d= & {} \sqrt{\frac{4P_{pp}}{\pi }}. \end{aligned}$$

### Determination of the centerlines

The center curve calculation algorithm was used to determine a single central curve of the vessel between the indicated vessel cross-sections defined in the previous step. The curve was driven by points representing the local maxima of the minimum distance from the edge of the $M_{OOBM}$ model, in the cross-sectional area perpendicular to the long axis of the vessel. The distance between subsequent points $\Delta$d was dependent on the $M_{OOBM}$ value at P, from where the next point was sought:

5 $$\begin{aligned} \Delta d= & {} M_{OOBM}(P)*f \end{aligned}$$

where f is the crawl rate. The typical f values for this study were in the range [0.5;1]. Values above 1, and especially above 2 in case of neighboring vessels could cause undesirable algorithm transitions between vessels. Values below 0.5 were impractical as they were generating very rough centerlines, especially for smaller vessels with radii close to study resolution.

### Calculation of the division zones of the vessel

The process of calculating division zones began with finding the point of decay of the central curves forming the division. The algorithm for each central curve forming the division found the last point for which the minimum distance from the second curve was less than the set value of d (assumed to be 0.2 mm—the spatial resolution of the auxiliary volumes). The split point was calculated as the geometric mean of the two points found. Its value was entered into the partition structure as a $T_0$.

Based on the location of the points found, the parameters of the partition zone were calculated. Split zone points were also used in further analysis of curvature and torsion (described in the following paragraph). The last steps in the division zone calculation were to determine whether it describes the actual division of the MCA or whether the aneurysm was removed—one of the component curves is the curve to the aneurysm. Of the identified zones, the one for which the split point was the furthest from the origin of the MCA trunk was chosen. This meant that it was a division zone describing the departure of the aneurysm dome from the parent vessel. The second curve of this zone was the curve of the parental aneurysm vessel. In this way, the aneurysm stem was identified. After this step, due to the scope of the analysis, zones other than those describing the actual division of the MCA were rejected.

### Description of the centerlines

As in the case of vessel cross-section sections, a number (type of curve) identifying the type of vessel described by it was assigned to further identify each curve. For example, during measurements of MCA divisions, the curve of type 1 was a curve to the aneurysm sac, the curves of successive branches forming the MCA obtain consecutive umbers 2, 3 and so on. Additionally it was possible to mark up to 25 control points along the curve. These point were used during algorithms assessment phase in true CTA studies. This functionality was implemented by introducing editable control points in the view (both MPR and 3D) of the central curve.

### Mathematical analysis of the centerlines

The assumption of mathematical analysis of the central curves was to determine correlations between distribution of the curvature and the torsion and evolution of vascular pathologies such as aneurysms and atherosclerosis. In order to calculate the curvature and the torsion of the center lines all curves were approximated with use of the Bezier curves. In order to partially decompose the crankshaft curve, it was decided to use the spline curves consisting of Bezier curves, connected at the site of the division of the vessel. The 6th grade curves and C3 continuity class were selected. The continuity class C3 was needed to ensure the continuity of the torsion function [20]. The 6th grade was a minimum grade allowing the creation of B-spline curves of any number of segments while maintaining this continuity class. The additional effect of the approximation with the parametric curves was the smoothing of the curves.

### Materials and validation method

**Fig. 7:**
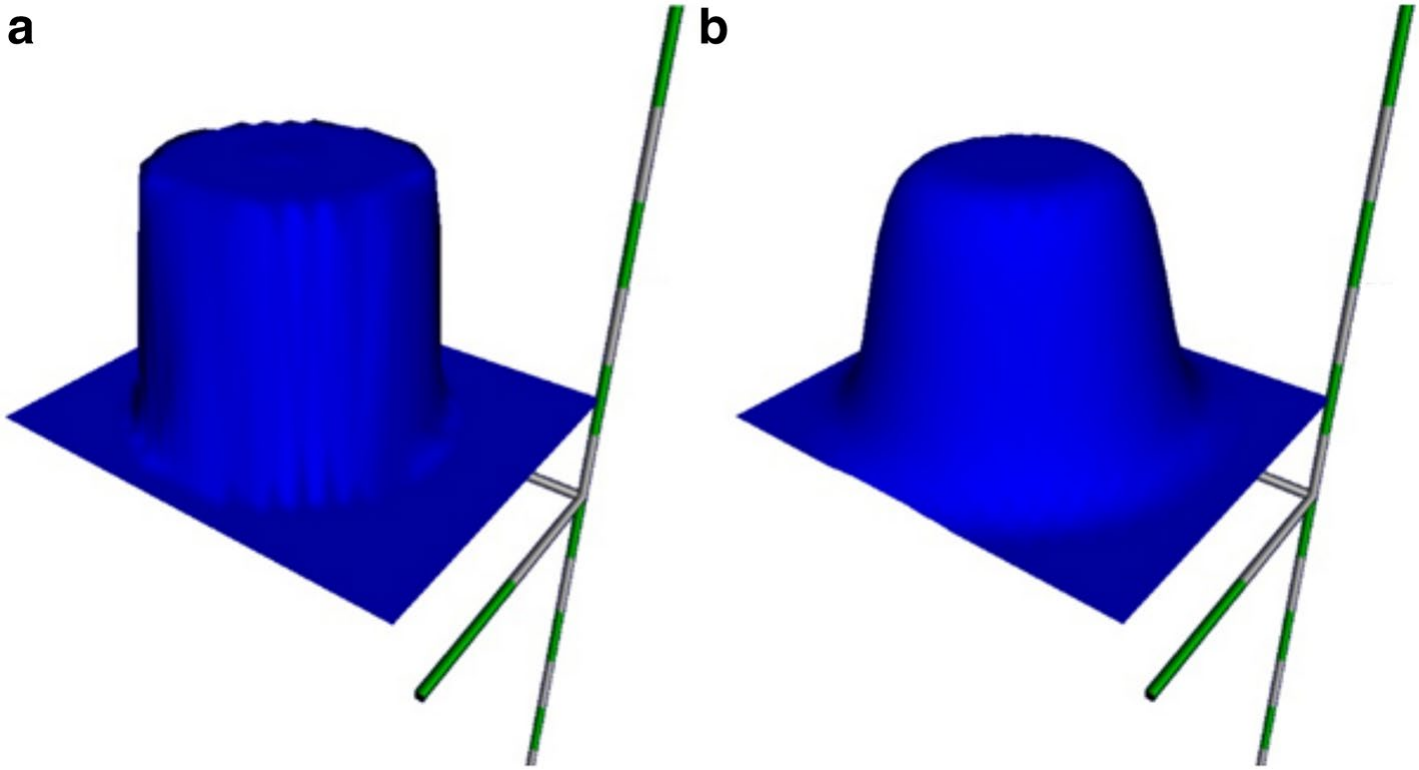
Function graphs HU(r): **a** C = 150, R = 5, a = 10, **b** C = 150, R = 5, a = 2

The purpose of this stage of the study was to assess the alignment and accuracy of the algorithms presented and to identify factors and conditions that could significantly impair the measurement values. Verification of the operation and accuracy of the algorithms presented above was made using synthetic models. Models were generated within a synthetic image data volume (SWD) dimension of $100 \times 100 \times 100\,{\text{mm}}$ and a 0.6 mm spatial resolution in all axes. The HU distribution on the vessel cross section was simulated by the formula:

6 $$\begin{aligned} HU(r)= & {} C+C\left(\frac{1}{2}-\frac{1}{1+e^{-\alpha (r-R)}}\right) \end{aligned}$$

where C—HU at the boundary of the vessel, r—distance from the center of the vessel for which we make the calculation, R—radius of the vessel, a—slope coefficient of the HU in the perimeter of the vessel. In Fig. 7 surface charts of HU(r) function values across the vessel are presented for different values of the parameters.

Models were drawn within a measuring volume using a spatial brush. The brush worked within a cube area of 1.5R sides and entered a WSP value in the SWD according to the above pattern if the calculated value was higher than the current one in the voxel. For all synthetic models the following parameters were used: C = 150, a = 10.

Experimental verification of the proposed methodology was conducted on 400 MCA trunk divisions examined with the contrast enhanced CTA. Patients were randomly assigned to this group from set of over 4500 individuals examined in the Computer Tomography Lab of the Second Department of Clinical Radiology Medical University of Warsaw between June 2006 and December 2016. For the measurements step 423 studies were assigned, but 23 (5.4$\%$) of them were rejected due to issues concerning study: mixed arterio-venous phase or insufficient contrast enhancement of the vessels.

## Results

### Validation of the algorithms

Within the volume, 1359 synthetic figures were generated: 381 torus and 978 spirals. Each of the examined figures had different shape parameters expressing the simulated vessel diameter (D), the basal radius of the vessel (R1), and spiral addition (R2). The parameter ranges used for generation of models are summarized in Table 1.

**Table 1:** Range of parameters used for generation of synthetic figures for validation of the division zones of the vessel algorithm

Model	$D_{min}$	$D_{max}$	$D_{step}$	$R1_{min}$	$R1_{max}$	$R1_{step}$	$R2_{min}$	$R2_{max}$	$R2_{step}$
Torus	1	5	0.25	5	30	1	Nd	Nd	Nd
Spiral	1.5	4	0.25	5	30	1	5	9	1

Patterns that overlap synthetic vessels were rejected, those in which R1 < 2D or R2 < 2D.

### Vessel diameter measurements validation

**Fig. 8:**
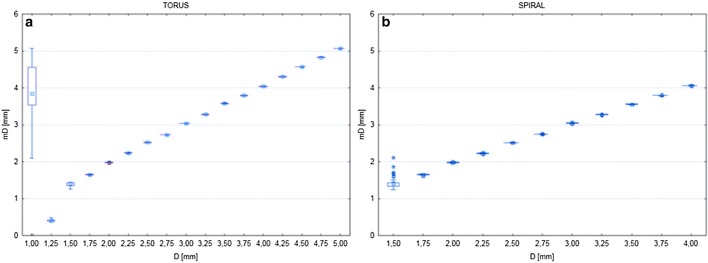
Graphical presentation of the relation between measured (mD) and true vessel model diameter (D) for toroidal (**a**) and spiral (**b**) models

On each model, at 10 random points ($M_{min}$ = 150, $O_{dv}$ = 160, $C_{min}$ = 170, and $C_{max}$ = 1500) vessel diameter measurements were performed and recorded in the file along with model parameters. For each measurement mean D, standard mean deviation and root mean square (RMS) were calculated. The average calculated parameters for all measurements separately for toroidal and spiral models are summarized in Table 2 and in Fig. 8. The presented data show accuracy of diameter and cross-section area of the vessel measurements.

**Table 2:** Summarized results of vessel diameter measurements validation

Parameter	Torus N	Torus mean	Torus min	Torus max	Torus std dev
$SD_D$	381	0.03	0.00	0.33	0.051
$SD_P$	381	0.10	0.00	0.74	0.12
$RMS_D$	381	0.12	0.017	1.00	0.21
$RMS_P$	381	0.35	0.039	1.10	0.26

Parameter	Spiral N	Spiral mean	Spiral min	Spiral max	Spiral std dev
$SD_D$	978	0.058	0.0070	1.29	0.12
$SD_P$	978	0.19	0.032	0.90	0.24
$RMS_D$	978	0.082	0.013	1.43	0.13
$RMS_P$	978	0.31	0.058	1.48	0.29

The results showed in Table 2 present very good mean $RMS_D$ and $RMS_P$ . In case of RMSD almost one order of magnitude lower than study resolution. For the same set of data, the maximal $RMS_D$ was more than twice bigger than this resolution. The cause of this discrepancy was found in Fig. 8.

This analysis shows very good measurement accuracy for vessels with D > 1.5 mm. Below this value the accuracy of measurement is clearly decreasing, breaking down for D = 1 mm. The result is consistent with the predictions based on the way in which the measurement algorithms and the spatial resolution of the synthetic data volume operate.

### Validation of the algorithm for determining the position of the points of the centerlines

The evaluation of the precision of the algorithm in determining the points of the central curves was based on the evaluation of the mean square distance of the point determined from the actual position described by the mathematical function. Within the synthetic volume, 36 figures were generated: 25 toruses and 11 spirals. The parameters describing the synthetic figures are summarized in Table 3.

**Table 3:** Summary of the range of parameters used for generation of synthetic models in the process of validating the accuracy of the center curves

Model	$D_{min}$	$D_{max}$	$R1_{min}$	$R1_{max}$	$R2_{min}$	$R2_{max}$
Torus	0.6	2	5	25	Nd	Nd
Spiral	1	2	5	30	8	100

**Fig. 9:**
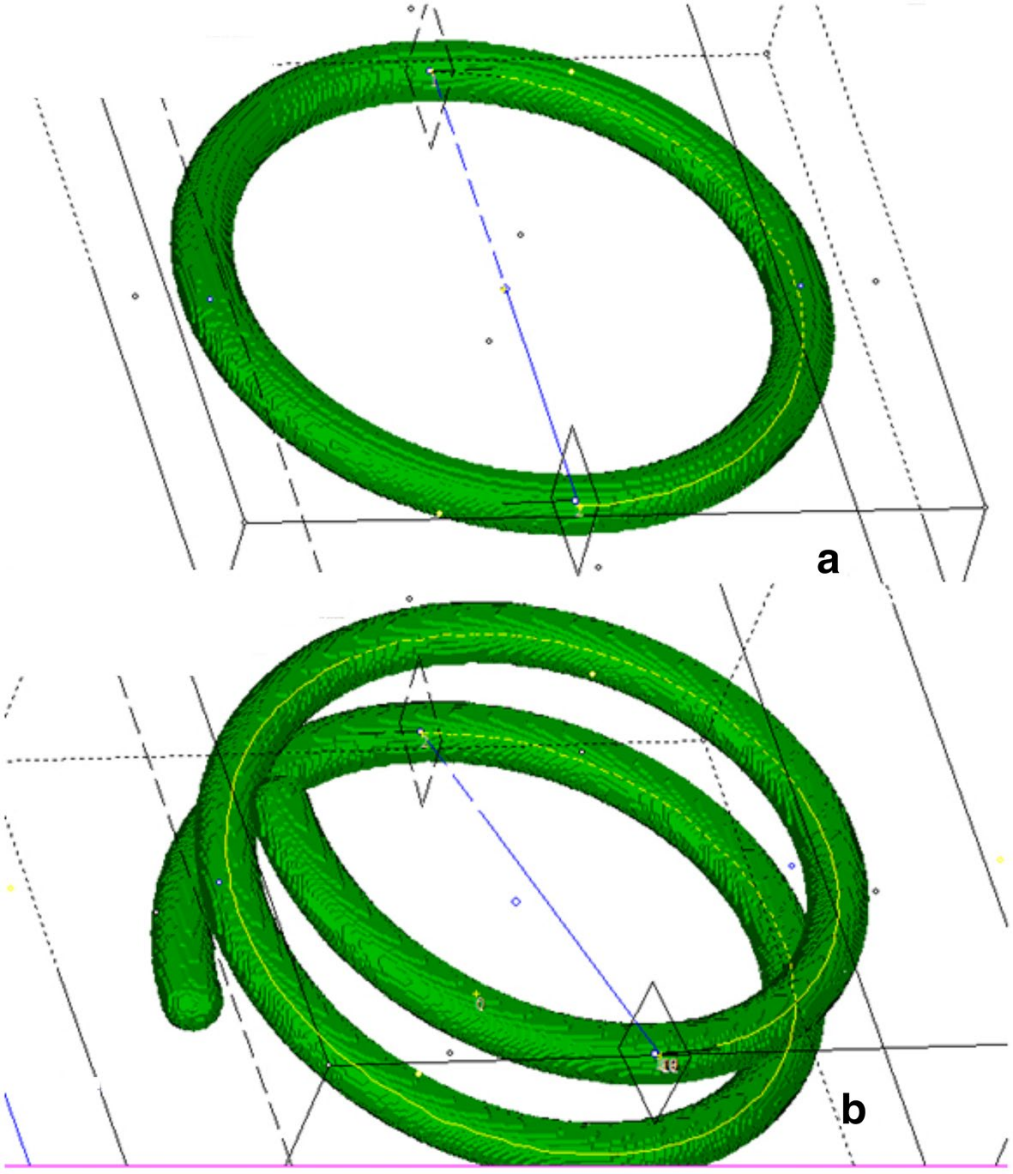
Validation of the center curves algorithm. Synthetic curves: torus (**a**), helix with center curves (yellow lines) between the measuring planes (**b**)

**Fig. 10:**
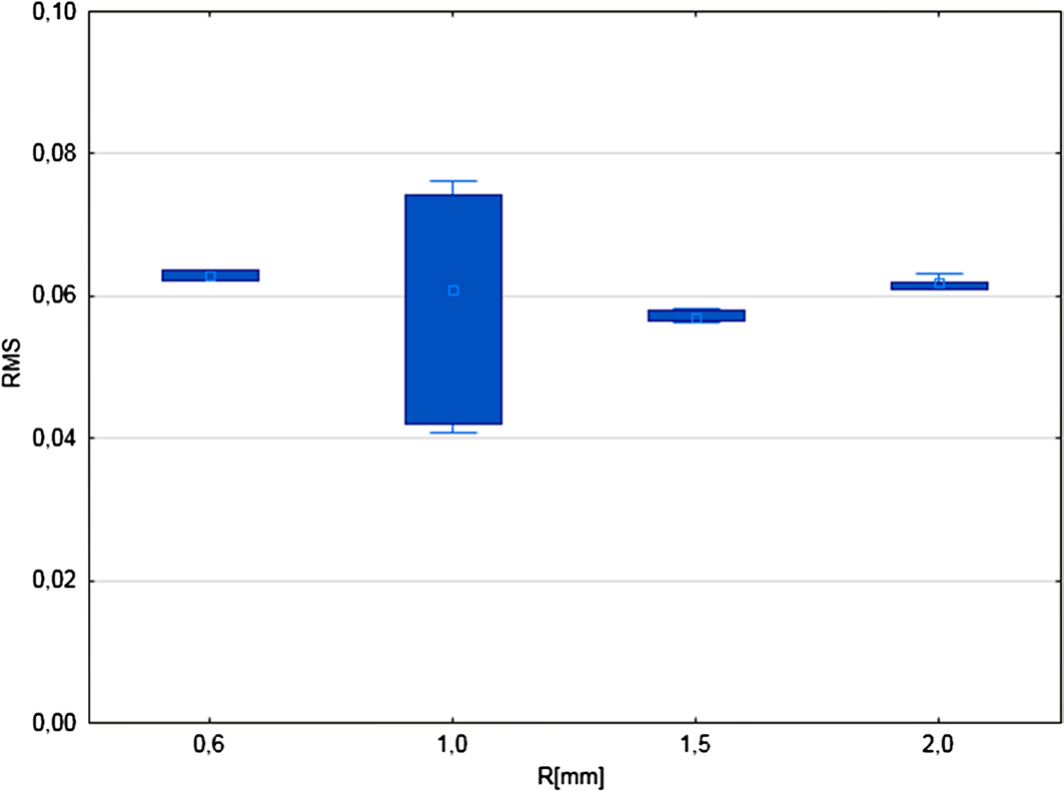
RMS values of diameter measurements grouped by radius of artificial vessels. Squares represents median values, boxes ranges of 25–75$\%$ percentiles and whiskers range of values

Within each model, a minimum of two central curves were calculated using algorithm 1 for dA = 3 / 2$\pi$ and *df* = 0.5 (Fig. 9). The obtained curves along with the model type (HELIX,TORUS) and its parameters are stored in separate files. For each generated central curve, the RMS was calculated in relation to the ideal mathematical model. The results are summarized in Table 4 and Fig. 10.

**Table 4:** Calculated RMS of precision of the points of the centerline positioning, separately for helix and torus models

Group	N	$RMS_{mean}$	$RMS_{median}$	$RMS_{min}$	$RMS_{max}$	$RMS_{SD}$
Helix	11	0.063	0.062	0.059	0.069	0.0034
Torus	25	0.061	0.062	0.041	0.076	0.0090

The obtained data showed very good accuracy of the positioning of the points of the central curves by the developed algorithms. No significant RMS L differences were found for the type and model parameters.

### Validation of the vessel bifurcation zone calculation

**Fig. 11:**
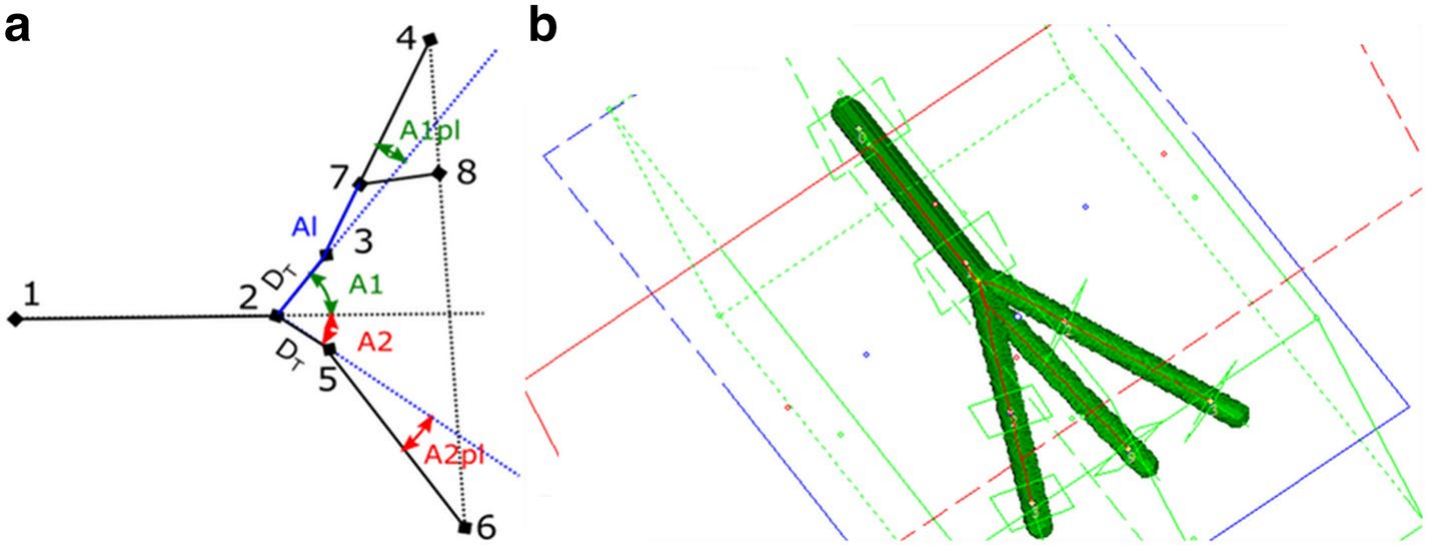
**a** Schema of artificial bifurcation zone. **b** Graphical presentation from validation process

The process of the validation of the bifurcation zone calculation was conducted utilizing artificial bifurcation zones (ABZ) with known BA, $VA_s$ and $C_oI_s$. Figure 11 shows artificial bifurcation zone schema and example of artificial model. Ranges of ABZ variables’ values are presented in Table 5. The ranges of these values were stated on the basis of the anatomical literature concerning brain vasculature [19, 21–23] and experience of our team gained during more than 8000 digital subtraction angiographies of the brain vasculature.

**Table 5:** The ranges of the values of variables of ABZ used during algorithms validation process

Parameter	Min	Max
A1	15	80
A2	15	80
$A1_{pl}$	− 15	15
$A2_{pl}$	− 15	15
$_wA_n$	0	0.15
$D_T$	3.5	4.5
$D_1$	2.5	4.2
$D_2$	2.0	3.0
$D_{an}$	2	6

The results presented as absolute difference between expected and measured value for selected variables are presented in Table 6. Average, absolute difference between real and measured angles were lower than 3.4°. Best results (below 1.9) were observed for dominant branch. When maximal absolute difference were assessed the worst results oscillated around 13° for BA and VA of no dominant branch. For dominant branch it was much better—below 7°.

For $C_oI$ average and maximal absolute differences were lowest for parent truck ($C_oI_P$) slightly over 0.008 and 0.05 respectively comparing to almost 0.04 and 0.15 for both branches.

**Table 6:** Results of bifurcation zone validation process as average, median, extremes and standard deviation of absolute differences between expected and measured values

Parameter	N	Average $\Delta$	Median $\Delta$	Min $\Delta$	Max $\Delta$	SD $\Delta$
$|\Delta BA|$	70	3.33	2.61	0.010	13.44	2.73
$|\Delta BA|$	70	3.33	2.61	0.010	13.44	2.73
$|\Delta VA_{dom}|$	70	1.83	1.47	0.030	6.58	1.43
$|\Delta VA_{ndom}|$	70	3.27	2.98	0.093	12.93	2.49
$|\Delta C_oI_{dom}|$	70	0.033	0.027	0.0006	0.12	0.026
$|\Delta C_oI_{ndom}|$	70	0.039	0.035	0.0002	0.15	0.033
$|\Delta C_oI_P|$	70	0.0085	0.0068	0.0002	0.054	0.0081

### The measurements process validation on clinical data

The measurements process validation of clinical data was performed for the middle cerebral artery division. The vessel level-0 was defined as MCA M1 segment (trunk), and level-1 MCA M2 (branches of the first division). Total number of 400 MCA divisions (M = 200) were measured and digitally described. According to Rhoton three main types of the MCA division were identified: bifurcation (BIF), multiple trunks (MT) and trifurcation (TRIF) [19] (Table 7).

**Table 7:** Number and percentage of the main three types of MCA division with divide into male and female and percentage of male and female divisions in each type of division

Division type	Number	Percent	Male	Female
Bifurcation	340	85.0	174 (51.2$\%$)	166 (48.8$\%$)
Multiple trunks	34	6.0	13 (54.2$\%$)	11 (45.8$\%$)
Trifurcation	36	9.0	13 (36.1$\%$)	23 (63.9$\%$)

For further geometrical analysis only dichotomous divisions: BIF and MT were selected which could be properly, geometrically described by previously defined parameters. TRIFs were rejected because in our opinion they could not be simply described as two combined bifurcations and need much more, also topological parameters which exceeds the scope of the presented paper.

The Shapiro–Wilk tests showed normal distribution only for vessel dimensions. The rest of the analyzed parameters presented with distribution different than normal.

According to the results of normal distribution test observed values of analyzed parameters with p-values of statistical tests comparing BIFs and M9Ts are presented in Tables 8 and 9.

**Table 8:** Median values and 25–75$\%$ percentile range of analyzed geometrical parameters for bifurcations and multiple trunk vessels divisions with p values for U Mann–Whitney test comparing both groups

Variable	Median values bifurcations	Median values multiple trunks	p (U Mann–Whitney test)
BA (degree)	82.8 (68.0–104.7)	91.5 (72.6–99.0)	$>0.4$
$VA_{dom}$ (degree)	46.0 (33.9–59.1)	48.7 (26.2–62.8)	$>0.8$
$VA_{ndom}$ (degree)	55.7 (42.1–71.3)	59.9 (51.5–80.0)	$>0.1$
$CoI_{dom}$	0.877 (0.785–0.943)	0.886 (0.822–0.974)	$>0.3$
$CoI_{ndom}$	0.877 (0.782–0.945)	0.806 (0.764–0.935)	$>0.3$
$CoI_T$	0.895 (0.820–0.949)	0.883 (0.803–0.953)	$>0.8$
$Kmax_{dom}$ (rad/mm)	0.246 (0.173–0.335)	0.258 (0.159–0.449)	$>0.5$
$Kav_{dom}$ (rad/mm)	0.151 (0.115–0.205)	0.187 (0.097–0.268)	$>0.2$
$Tav_{dom}$ (rad/mm)	0.321 (0.239–0.454)	0.334 (0.213–0.432)	$>0.5$
$Kmax_{ndom}$ (rad/mm)	0.308 (0.185–0.450)	0.381 (0.298–0.640)	$<0.05$
$Kav_{ndom}$ (rad/mm)	0.177 (0.125–0.236)	0.216 (0.163–0.318)	$<0.05$
$Tav_{ndom}$ (rad/mm)	0.386 (0.281–0.525)	0.440 (0.318–0.549)	$>0.4$

**Table 9:** Average values and SD of vessels average diameters for bifurcations and multiple trunks with p values for t-test comparing both groups

Variable	Average values bifurcations	Average values multiple trunks	p (t-test)
$D_T$ (mm)	2.9 ± 0.55	2.8 ± 0.51	$>0.5$
$D_{dom}$ (mm)	2.4 ± 0.41	2.4 ± 0.56	$>0.6$
$D_{ndom}$ (mm)	1.8 ± 0.41	1.5 ± 0.43	<* 0.001*

Statistically significant differences were italicized

The significant differences between both groups concern $Kmax_{ndom}$ and $Kav_{ndom}$ as well as diameters of non-dominant branches. Both Kmax and Kav presented higher values in MT group comparing to BIFs: 0.381 rad/mm (0.298–0.640) vs. 0.308 rad/mm (0.185–0.450) and 0.216 rad/mm (0.163–0.318) vs. 0.177 rad/mm (0.125−0.236) respectively.

DT was higher (2.87 mm ± 0.55 vs. 2.79 mm ± 0.51) and Ddom lower (2.37 mm ± 0.41 vs. 2.41 mm ± 0.56) in BIFs comparing to MTs.

$D_{ndom}$ was higher (1.8 mm ± 0.41 vs. 1.5 mm ± 0.43) in BIFs comparing to MTs.

In the next step the parameters were correlated with age. Process of correlation was conducted utilizing Spearman coefficient, separately for each type of division. Additional comparison of significance of differences between both groups were performed. Results are presented in Table 10. For BIF type of division multiple, significant correlations with age were found for variables: BA, VA, $CoI_T$, $Kav_{dom}$, $Tav_{dom}$ and D. Most of these correlations presented very low p values. For MT type of divisions statistically significant correlations were found for Kmax and Kav values of dominant branch. Form most parameters signs of R coefficients were same for both types of divisions. For BIF type of divisions much lower values of p were observed for lower values of R coefficient comparing to MT. This situation was caused by ten times higher number of BIF type of divisions (n = 340 vs. n = 34) and specificity of p calculation for R coefficients.

**Table 10:** Spearman’s correlations coefficients of analyzed variables with age for divisions of type BIF and MT with specified p values of significance test

Parameter	BIF R	BIF p	MT R	MT p
BA	0.1940	0.0003	0.1836	0.3905
$VA_{dom}$	0.1378	0.0109	0.0209	0.9228
$VA_{ndom}$	0.2092	0.0001	0.1866	0.3825
$CoI_{dom}$	− 0.0285	0.6006	0.1806	0.3985
$CoI_{ndom}$	− 0.1023	0.0596	0.1179	0.5832
$CoI_{T}$	− 0.1604	0.0030	-0.3011	0.1528
$Kmax_{dom}$	0.1132	0.0369	0.4172	0.0425
$Kav_{dom}$	0.1526	0.0048	0.4333	0.0344
$Tav_{dom}$	− 0.1489	0.0059	0.1310	0.5419
$Kmax_{ndom}$	0.0979	0.0715	− 0.0191	0.9293
$Kav_{ndom}$	0.0921	0.0898	0.0379	0.8606
$Tav_{ndom}$	− 0.2263	0.0000	− 0.0526	0.8070
$D_{T}$	0.1713	0.0015	− 0.2001	0.3484
$D_{dom}$	0.1856	0.0006	− 0.2358	0.2673
$D_{ndom}$	0.1557	0.0040	− 0.2315	0.2765

Significant (p < 0.05) correlations are italicized

**Fig. 12:**
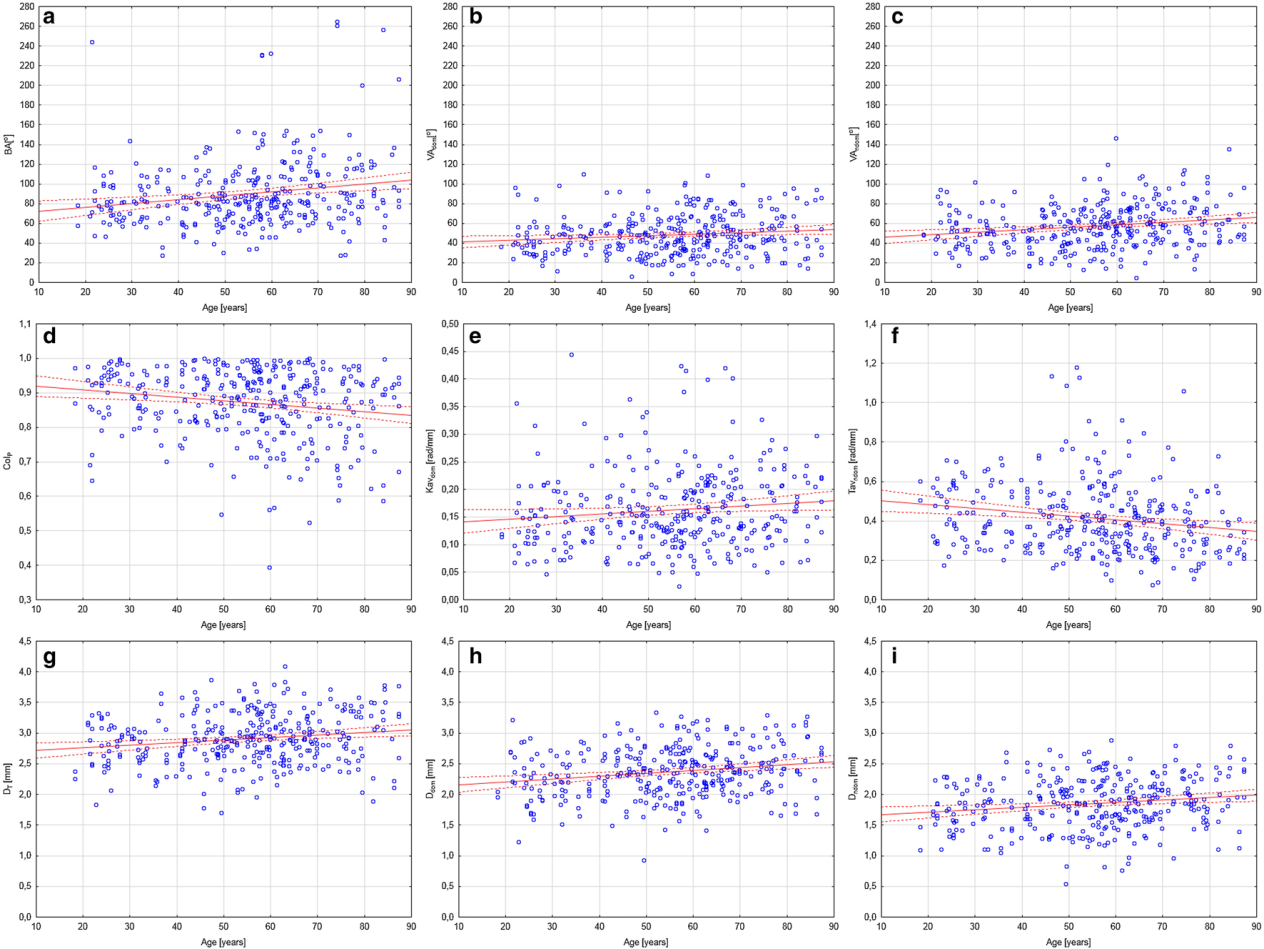
Scatter plots of selected parameters presenting significant correlations with age. **a** BA; **b** $VA_{dom}$; **c** $VA_{ndom}$; **d** $CoI_P$; **e** $Kav_{dom}$; **f** $Tav_{ndom}$; **g** $D_T$; **h** $D_{dom}$; **i** $D_{ndom}$. Solid line represents trend line. Dotted lines represents confidence intervals of 95$\%$

Scatter plots of the selected parameters presenting significant correlations with age could be found in Fig. 12. The general impression from presented plots is that for VA and vessel diameter we can observe much more narrow, and constant value ranges than the rest, especially CoI. In case of BA, Kmax, Tav and CoI despite observed trends we can observe widening of values range with age.

## Discussion

Geometrical and mathematical description of vessel geometry was addressed in current literature for a few years. Its linkage to vessel flow was confirmed in many studies [4–7, 24–27]. Most of presented systems, contrary to ours consisted of many separated modules for preprocessing, segmentation, measurement and post-processing of the data. The differences also include different way of vessel models representation and consequently way of centerlines determination. Now we discuss limitations of presented methods, results of algorithm validation on artificial models and finally on native, medical CTA data.

### Main features of vessel segmentation and centerline detection

In the presented study, opposite to works of Italian [11–16, 24] and Irish [17] groups vessels models were defined not as 3D meshes but rather group of voxels of the same index values.

The Italian group after segmentation transformed vessel models into 3D meshes and centerlines were determined utilizing Voronoi diagrams of these meshes. In this technique centerline points in vessel cross section are point maximally distant from model surface [11–17, 28, 29]. This idea of maximal distance from model surface is similar to the assumption in the proposed algorithm. Both techniques should also be resistant to errors due to close relationship between two vessels. In this case both generate two local maxima, each in geometrical center of abutting vessels.

Methodology of centerline detection of the Irish group was not clearly described [17]. Only short information, that CLs were lead over geometrical centers of vessel cross sections, is available. This tactic could cause errors in mentioned regions of abutting vessels.

The presented strategy for segmentation is very simple but sufficient as preprocessing for created algorithm for CLs.

### Mathematical analysis of the CLs

The basic concept of CLs transformation into mathematical functions was the smoothing and determination of curvature and torsion. This step was realized by transformation into Bezier splines of degree 6 and continuity of $C^{3}$ degree [20] was proposed and distribution of control points was dependent on local radius. That was different comparing to the works of Italian and Irish groups.

Transformation into mathematical function allows to avoid indetermination of the curvature and the torsion of CL which could occur in straight vessel segments when using discrete method. The group of OFlynn used transformation into 9th degree polynomials, and Antiga’s group utilizes discrete method, as it implies from documentation of the Vascular Modelling Toolkit.

The first advantage of the first approach is that process of approximation additionally cause curve smoothing and allows for easy and continuous calculation of curvature and torsion. The first approach disadvantage is that polynomial could precisely describe only relative small segment of the vessel. Increasing degree of the polynomial increases the length of approximated CL but causes problems with locality approximation. It is also difficult to dynamically change degree of fitting to avoid over and under approximation where vessel changes it diameter.

In the second approach both problems are solved, because separation of CLs points depends on local radius and CL could have any length. There are two disadvantages: before Frénet-Serret frame calculation it is necessary to smooth the curve, which causes known problems with shape preservation, setting proper level of noise filtering [30, 31]. The second, discrete algorithm for Frénet frames calculation could fail on straight portions of vessels: the frame could be indefinable or there could be fast and random fluctuations of its orientation, which causes incorrect calculation of the curvature and torsion.

The approach proposed in this study seems to solve all mentioned problems. Bezier segment degree is chosen to allow construction spline of any number with preservation continuity of third derivative [20]. Approximation within each segment is local. Degree of fitting could be altered by setting control points in distances depending on local vessel diameter.

### Vessels diameter measurements

The first limitation was identified during process of measurements validations on artificial models. As presented in Table 2 and in Fig. 8 vessel diameter measurements performs very well down to about 2 * voxel size, which means that measurement of vessels less than this value is not reliable. This issue is a consequence of Nyquist–Shannon sampling theorem. Above this limit presented method reaches subvoxel accuracy of measurements.

### Bifurcation zone calculations

Validation of vessel bifurcation zone presented in Table 6, showed very good accuracy of measurements of presented algorithms.

### Application of the algorithms to clinical data

The most important step was analysis of clinical data performed on native CTA images of patients. We have observed that the software allows for all measurements to be performed if proper (arterial) or slightly delayed phase of the study was available. In case of mixed arterio-venous phase CLs estimation was possible but required extensive user interactions, so these cases were rejected from calculations.

The percentage representation of various MCA divisions types was similar to presented in anatomical study of Rhoton [19]. The main type of division was BIF consisting of 85$\%$ of divisions, the second was TRIF (9$\%$) and the last frequent MT (6$\%$). In the Rhoton’s study these percentages are as follows: 78$\%$, 12$\%$ and 10$\%$ respectively. In each group almost equal numbers of male and female divisions were analyzed (Table 7).

Created application allowed for analysis of 400 MCA divisions. Acquired data and metadata were stored in local database and could be used in the future. In first step, the value distribution analysis, it was showed that almost all parameters had distribution different from normal. In the second step, median and average values of analyzed parameters were calculated for BIF and MT type of division. There were found significant correlations for 9 of analyzed variables with patients age utilizing Spearman correlation coefficients. It was found that BA, VA, $Kav_{dom}$ and D increases their values with patients age. Contrary $CoI_{P}$ and $Tav_{ndom}$ decreases their values. Further analyzes, especially scatter plots showed that additional widening of Kav, CoI and Tav values range with age. This observation is similar to the widening of CCA bifurcation angle with age reported by Thomas [16].

## Conclusions

This article presents a concept of integrated system for measurements and analysis of large amount of vascular and anatomical data. The system implements most precise methods for vascular anatomy description based on centerlines. Use of spline curve consisting of Bezier segment for centerlines approximation is a new concept. Unlike other reported solutions all proposed methodology steps were integrated which allows the analysis of variability of geometrical parameters in a large number of MCA bifurcations using only one application This allows for determination of significant trends in the parameters variability with age and differences between MCA division types.

Splines of 6-th degree and continuity of C3 degree allow unlimited length approximation of the vessels centerlines. Separation of CL spline control points was set on local vessel radius which assures proper vessel geometry approximation without risk of oversampling. Taking into account the obtained results full automation of proposed methodology allows in the future to analyze large amount of medical records and use artificial intelligence for deeper analysis and support for risk stratification for vascular lesions such as aneurysms and atherosclerosis in any vascular territory.

## References

[1] Nixon AM, Gunel M, Sumpio BE (2010). The critical role of hemodynamics in the development of cerebral vascular disease. J Neurosurg.

[2] Kleinstreuer C, Hyun S, Buchanan JR, Longest PW, Archie JP, Truskey GA (2017). Hemodynamic parameters and early intimal thickening in branching blood vessels. Crit Rev Biomed Eng.

[3] Kleinstreuer C, Hyun S, Buchanan JR, Longest PW, Archie JP, Truskey GA (2001). Hemodynamic parameters and early intimal thickening in branching blood vessels. Crit Rev Biomed Eng.

[4] Wells DR, Archie JP, Kleinstreuer C (1996). Effect of carotid artery geometry on the magnitude and distribution of wall shear stress gradients. J Vasc Surg.

[5] Niu L, Meng L, Xu L, Liu J, Wang Q, Xiao Y, Qian M, Zheng H (2015). Stress phase angle depicts differences in arterial stiffness: phantom and in vivo study. Phys Med Biol..

[6] Niu L, Zhu X, Pan M, Derek A, Xu L, Meng L, Zheng H (2018). Influence of vascular geometry on local hemodynamic parameters: phantom and small rodent study. BioMed Eng Online.

[7] Chiastra C, Iannaccone F, Grundeken MJ, Gijsen FJH, Segers P, De Beule M, Serruys PW, Wykrzykowska JJ, van der Steen AFW, Wentzel JJ (2016). Coronary fractional flow reserve measurements of a stenosed side branch: a computational study investigating the influence of the bifurcation angle. BioMed Eng Online.

[8] Thompson BG, Brown RD, Amin-Hanjani S, Broderick JP, Cockroft KM, Connolly ES, Duckwiler GR, Harris CC, Howard VJ, Johnston SC, Meyers PM, Molyneux A, Ogilvy CS, Ringer J, Torner J. Guidelines for the management of patients with unruptured intracranial aneurysms: a guideline for healthcare professionals from the American Heart Association/American Stroke Association; 2015. 10.1161/STR.0000000000000070 26089327

[9] Steiner T, Juvela S, Unterberg A, Jung C, Forsting M, Rinkel G (2013). European stroke organization guidelines for the management of intracranial aneurysms and subarachnoid haemorrhage. Cerebrovasc Dis.

[10] Wardlaw JM, White PM (2000). The detection and management of unruptured intracranial aneurysms. Brain.

[11] Antiga L, Steinman DA (2004). Robust and objective decomposition and mapping of bifurcating vessels. IEEE Trans Med Imag.

[12] Lee SW, Antiga L, Spence JD, Steinman DA (2008). Geometry of the carotid bifurcation predicts its exposure to disturbed flow. Stroke.

[13] Antiga L. Patient-specific modeling of geometry and blood flow in large arteries. Politecnico di Milano; 2002.

[14] Piccinelli M, Veneziani A, Steinman DA, Remuzzi A, Antiga L (2009). A framework for geometric analysis of vascular structures: application to cerebral aneurysms. IEEE Trans Med Imag.

[15] Piccinelli M, Bacigaluppi S, Boccardi E, Ene-Iordache B, Remuzzi A, Veneziani A, Antiga L (2011). Geometry of the internal carotid artery and recurrent patterns in location, orientation, and rupture status of lateral aneurysms: an image-based computational study. Neurosurgery.

[16] Thomas JB, Antiga L, Che SL, Milner JS, Steinman DA, Spence JD, Rutt BK, Steinman DA (2005). Variation in the carotid bifurcation geometry of young versus older adults: implications for geometric risk of atherosclerosis. Stroke.

[17] O’Flynn PM, O’Sullivan G, Pandit AS (2007). Methods for three-dimensional geometric characterization of the arterial vasculature. Ann Biomed Eng.

[18] Rhoton AL (2002). The cerebral veins. Neurosurgery.

[19] Rhoton AL (2002). The supratentorial arteries. Neurosurgery.

[20] Hagen H (1986). Bezier-curves with curvature and torsion continuity. Rocky Mt J Math..

[21] Elsharkawy A, Lehecka M, Niemela M, Billon-Grand R, Lehto H, Kivisaari R, Hernesniemi J (2013). A new, more accurate classification of middle cerebral artery aneurysms: computed tomography angiographic study of 1,009 consecutive cases with 1,309 middle cerebral artery aneurysms. Neurosurgery.

[22] Rhoton AL (2000). The cerebellar arteries. Neurosurgery.

[23] Rhoton AL (2002). Aneurysms. Neurosurgery.

[24] Passerini T, Sangalli LM, Vantini S, Piccinelli M, Bacigaluppi S, Antiga L, Boccardi E, Secchi P, Veneziani A (2012). An integrated statistical investigation of internal carotid arteries of patients affected by cerebral aneurysms. Cardiovasc Eng Technol.

[25] Fan J, Wang Y, Liu J, Jing L, Wang C, Li C, Yang X, Zhang Y (2015). Morphological-hemodynamic characteristics of intracranial bifurcation mirror aneurysms. World Neurosurg.

[26] Xu J, Yu Y, Wu X, Wu Y, Jiang C, Wang S, Huang Q, Liu J (2013). Morphological and hemodynamic analysis of mirror posterior communicating artery aneurysms. PLoS ONE.

[27] Liu J, Xiang J, Zhang Y, Wang Y, Li H, Meng H, Yang X (2014). Morphologic and hemodynamic analysis of paraclinoid aneurysms: ruptured versus unruptured. J Neurointerv Surg.

[28] Hassan T, Timofeev EV, Saito T, Shimizu H, Ezura M, Matsumoto Y, Takayama K, Tominaga T, Takahashi A (2005). A proposed parent vessel geometry-based categorization of saccular intracranial aneurysms: computational flow dynamics analysis of the risk factors for lesion rupture. J Neurosurg.

[29] Ramachandran M, Retarekar R, Harbaugh RE, Hasan D, Policeni B, Rosenwasser R, Ogilvy C, Raghavan ML (2013). Sensitivity of quantified intracranial aneurysm geometry to imaging modality. Cardiovasc Eng Technol.

[30] Pastva TA. Bezier curve fitting. Ph.D. Thesis; 1998.

[31] Mansouryar M, Hedayati A (2012). Smoothing via iterative averaging (sia) a basic technique for line smoothing. Int J Comput Elec Eng.

